# ZIKV Envelope Domain-Specific Antibodies: Production, Purification and Characterization

**DOI:** 10.3390/v11080748

**Published:** 2019-08-13

**Authors:** Sami Akhras, Marie-Luise Herrlein, Fabian Elgner, Thomas Holzhauser, Eberhard Hildt

**Affiliations:** 1 Department of Virology, Paul-Ehrlich-Institut, 63225 Langen, Germany; sami.akhras@pei.de (S.A.); marie-luise.herrlein@pei.de (M.-L.H.); fabian.elgner@pei.de (F.E.); 2 Department of Allergology, Paul-Ehrlich-Institut, 63225 Langen, Germany; thomas.holzhauser@pei.de; 3 German Center for Infection Research (DZIF), 38124 Braunschweig, Germany

**Keywords:** Zika virus, envelope protein, polyclonal antibody, non-/weak-neutralizing antibodies, epitopes

## Abstract

Infection with Zika virus (ZIKV) came first to public attention after it was found to be associated with congenital microcephaly during the outbreak in Brazil (2015–2016). Diagnosis of ZIKV suffers from extensive cross-reactivity with other *Flaviviruses*, which are circulating in many ZIKV epidemic areas. Due to the fatal outcome of ZIKV infection during pregnancy, detailed knowledge about neutralizing and non-neutralizing epitopes is crucial for the development of robust detection systems of protective antibodies. Therefore, additional information about ZIKV immunogenicity and antibody response is required. In this project, we report the production, purification and characterization of six different polyclonal antibodies against ZIKV envelope (E) protein. The produced antibodies bind to isolated ZIKV E protein as well as to the surface of ZIKV particles, interestingly without being potently neutralizing. Surface plasmon resonance measurement showed that these antibodies bind with high affinity to ZIKV E protein. Epitope mapping revealed that the epitopes are distributed among the three ZIKV E domains with seven binding sites. These identified binding sites overlap only partially with the previously described epitopes recognized by neutralizing antibodies, which is in accordance with their lack of potent neutralizing activity. Additionally, these antibodies showed neither cross-reactivity nor potent neutralizing activity against West Nile virus, a related flavivirus. The gained set of data helps to extend our understanding about the distribution of neutralizing and non-/weak-neutralizing epitopes in ZIKV E protein, and provides a rationale for ZIKV vaccine design and development of robust detection assays for neutralizing antibodies.

## 1. Introduction

Zika virus (ZIKV) is a mosquito-borne virus, which was first isolated in 1947 from a rhesus monkey in the Zika forest in Uganda [1]. The first described infection of humans with ZIKV was in Nigeria in 1954 [2]. Before the first reported outbreak, which occurred in Yap Island in Micronesia (2007) [3], only 14 cases of human infection with ZIKV were documented. The outbreak in Yap Island was followed by a larger epidemic in French Polynesia in the South Pacific in 2013–2014 [4]. ZIKV started to attract international attention after being linked to some neurological complications in humans such as congenital microcephaly and the Guillain–Barré syndrome (GBS) [5,6,7]. During the ZIKV outbreak in Brazil (2015–2016) [8], the WHO declared the outbreak as a public health emergency of international concern (PHEIC, February 2016). ZIKV belongs to the genus *Flavivirus* of the family *Flaviviridae* and has a positive single stranded RNA genome, which is about 11,000 bases long. This genome encodes for three viral structural proteins (core protein: C, precursor of the membrane protein: prM, and the envelope protein: E) and seven non-structural proteins NS1, NS2A, NS2B, NS3, NS4A, NS4B, and NS5, which are necessary for the virus assembly and viral genome replication [9]. In the mature virions, the virus genome is surrounded by the capsid and the lipid membrane, in which 180 copies of each the E and the membrane (M) proteins are embedded to cover the entire ‘smooth’ surface of the virions. The envelope ectodomain consists of three domains: D1, D2, and D3 and is connected C-terminally via a stem segment to a transmembrane anchor. D1 is located in the center between D2 and D3 and comprises three segments: Amino acid (aa) 1–51, aa 132–192, and aa 280–295. This domain contains the unique glycosylation site in the ZIKV E protein: N154. The D2 domain (aa 52–131 and aa 193–279) contains the fusion loop (FL) (aa 98–109). The third domain (D3) displays an immunoglobulin-like module and comprises the residues 296–403 [10]. ZIKV E protein, like other *Flaviviruses* E proteins, represents a major target for neutralizing antibodies. Up to now, the crystal structures of more than 10 ZIKV neutralizing antibodies (or antibody fragments) in complex with ZIKV (or ZIKV E protein) have been resolved. Some of these antibodies show binding with relatively high affinities such as 2A10G6 (*K*_D_ = 2.7 nM, [10]), C8 (*K*_D_ = 9 ± 1 nM, [11]), ZK2B10 (*K*_D_ = 1.06 nM in pH 7.5, [12]), ZV-48 (*K*_D_ = 35 ± 0.8 nM, [13]), ZV-64 (*K*_D_ = 32 ± 13 nM, [13]), ZKA190 (*K*_D_ = 0.3 nM, [14]) and ZV-67 (*K*_D_ = 8.8 ± 1.7 nM, [13]), while others exhibit binding with relatively lower affinities such as A11 (*K*_D_ = 840 ± 470 nM, [11]), Z20 (*K*_D_ = 1.6 × 10^−7^ M, [15]), Z23 (*K*_D_ = 4.4 × 10^−7^ M, [15]), and Z3L1 (*K*_D_ = 4.39 x 10^−6^ M, [15]). Many of these antibodies (e.g., ZV2B10, ZV-48, ZV-64, ZV-67, Z006 [16], ZKA190, and Z021 [17]) bind to the D3 domain of the ZIKV E protein, while others bind to both D1 and D2 (e.g., Z3L1 and ZIKV-195 [18]), or to D2 (e.g., Z20). Each of ZIKV-117 [19], C8, C10 [20] and A11 antibodies were shown to recognize epitopes that span across the E dimer interface, while the flavivirus broadly-neutralizing antibody, 2A10G6, was found to bind an epitope in the fusion loop of ZIKV E protein. Despite the gained knowledge about the structure of ZIKV E protein and antibodies specifically binding to ZIKV E protein, the accurate diagnosis of ZIKV infection using serological tests still suffers from extensive cross-reactivity with other *Flaviviruses*, such as Dengue Virus (DENV), which might share the same epidemic areas with ZIKV [21]. Most importantly, at present there is no robust diagnostic test available that allows the specific detection of neutralizing antibodies. In addition, the antibody-dependent enhancement (ADE) effect might complex the process of developing vaccines against ZIKV. Therefore, the immunogenicity of ZIKV E protein, and the correlation between targeted epitopes and non-/weak-neutralization should be investigated in more detail.

In order to gain more information about distribution pattern of neutralizing and non-/weak-neutralizing epitopes in the ZIKV E protein, we present in this work the design, production, and purification of different ZIKV E protein domains, which we used for immunization and production of E-specific polyclonal antibodies. The produced antibodies were purified, characterized, and compared to the well-described ZIKV neutralizing monoclonal antibodies regarding their binding sites and neutralizing activity. It was found that the antibodies bind with high affinity ZIKV E domains with no potent neutralization activity, which indicates that their binding sites might contain non-/weak-neutralizing epitopes. In addition, these antibodies showed no reactivity against West Nile virus (WNV) E protein and no capacity to potentially neutralize WNV, which indicates the specificity of our antibodies, at least regarding WNV. This information may help the development of effective vaccines and more reliable assays to detect, not only ZIKV-specific, but also neutralizing and protective antibodies.

## 2. Materials and Methods

### 2.1. Construct Design

The coding sequence (Zika virus Strain PF13/251013-18-Asian, GenBank Sequence Accession KY766069) for either the truncated ZIKV E protein (aa 1 to 409), which lacks the stem region and the transmembrane domain, the ZIKV E domains 1+2 (aa 1 to 295), and the truncated ZIKV E Domain 3 (aa 296 to 409) were amplified by PCR and inserted in the modified plasmid vector pET21a-streptavidin-alive (Addgene #20860 [22], Addgene, MA, USA). The modification of the plasmid was done by removing a stop codon after the coding sequence of streptavidin-His-tag to enable the production of ZIKV E protein domains with streptavidin-His-tag as fusion proteins. The designed constructs allowed the fusion of another His-tag C-terminally to the inserted regions. The generated constructs were designated here as: streptavidin-ZIKV E, streptavidin-ZIKV ED1+2, and streptavidin-ZIKV ED3. Sequencing of the generated constructs revealed the correct sequence except of F198L and D230N substitution in the streptavidin-ZIKV ED1+2 and streptavidin-ZIKV E constructs, respectively. These constructs were used to produce the recombinant proteins for rabbit immunization. In addition, the PCR-amplified products were inserted directly in empty pET21a(+) vector using primers that allow the C-terminal fusion of strep-tag to the ZIKV E proteins. The generated constructs were designated here as: ZIKV E, ZIKV ED1+2, and ZIKV ED3, and were used for the polyclonal antibody purification and characterization.

### 2.2. Protein Production and Rabbit Immunization

Competent *E. coli* BL21[DE3], transformed with the cloned plasmids, were cultured at 37 °C in LB medium with shaking at 250 rpm. Induction was performed for 4-6 h by adding 1 mM IPTG (Thermo Fisher Scientific, Waltham, USA) at OD600 = 0.4–0.5. The induced cells were pelleted by centrifugation at 5000 *g* for 15 min at 4 °C and washed once with ice-cold PBS. The recombinant proteins were accumulated in the induced cells as inclusion bodies and were isolated and purified as following: The bacterial pellet from 2 L induction culture was resuspended in 10 mL lysis buffer: 100 mM Tris-HCl, pH 8, 250 µg/mL lysozyme (Carl Roth, Karlsruhe, Germany), 150 g/L sucrose, 10 µg/mL aprotinin, 25 µg/mL Leupeptin, 20 µg/mL pepstatin, 1 mM PMSF (AppliChem, Darmstadt, Germany), and 1 µL benzonase (Merck, Darmstadt, Germany). The lysate was incubated for 20 min at RT, followed by 4 × 15 s ultrasound cycles performed on ice. The lysate was then centrifuged at 2,0000 *g* for 20 min at 4 °C. The pellet was washed 3–5 times with washing buffer consisting of 100 mM Tris-HCl, pH 8, 2% Triton X-100 (Merck, Darmstadt, Germany), followed by washing with the same buffer supplemented with 1 M NaCl. Final wash step was performed using 100 mM Tris-HCl, pH 8. The purified inclusion bodies were resuspended for 1 h at RT in guanidine-HCl buffer consisting of 6 M guanidine-HCl, 100 mM Na_2_HPO_4_·2H_2_O, 10 mM Tris, pH 8 (Carl Roth, Karlsruhe, Germany) and centrifuged at 20,000 *g* for 30 min at 4 °C. For purification of the streptavidin-fusion proteins, Ni-NTA affinity chromatography columns (GE Healthcare, Munich, Germany) were used under denaturing conditions, followed by stepwise dialysis using PBS buffer containing decreasing concentrations of urea to replace the denaturing agent (urea) with PBS. For the purification of ZIKV E proteins which contain strep-tag, the resuspended inclusion bodies were directly subjected to stepwise dialysis using PBS buffer containing decreasing concentrations of urea, followed by purification using strep-tactin affinity columns (GE Healthcare, Munich, Germany) under native conditions. The identity and purity of the purified proteins were checked by western blotting and coomassie staining of SDS-gels. The purified and denatured streptavidin-fusion proteins were used for rabbit immunization (Seramun Diagnostica GmbH, Heidesee, Germany). Two rabbits were immunized with each protein (1–1.5 mg for each rabbit): Rabbits K89 and K90 were immunized with streptavidin-ZIKV E, K87 and K88 were immunized with streptavidin-ZIKV ED1+2, and K45 and K48 were immunized with ZIKV ED3. Pre- and hyper-immune sera were collected and applied for further experiments.

### 2.3. Antibody Purification

#### 2.3.1. Preparation of the Affinity Beads

For isolation of ZIKV E-specific antibodies from the sera, the strep-tagged ZIKV E proteins were coupled to NHS-activated sepharose beads (GE Healthcare, Munich, Germany) according to the manufacturer’s protocol. The beads were then filled in suitable columns, and the ÄKTA purifier system (GE Healthcare, Munich, Germany) was applied for purification of corresponding sera as shown in Table 1:

#### 2.3.2. Antibody Purification

The collected sera were filtered and 5–15 mL were applied on the column for each purification. After washing with PBS, the attached antibodies were eluted by 0.1 M glycine.HCl (Carl Roth, Karlsruhe, Germany), pH 2.7, followed by direct pH neutralization of the eluted antibodies using 1 M Tris-HCl (Carl Roth, Karlsruhe, Germany), pH 8.5. The purity of the eluted antibodies was checked by Coomassie staining of SDS-gels.

### 2.4. SDS-PAGE and Western Blot Analysis

Infected or uninfected human epithelial lung carcinoma cells (A549) and African green monkey kidney cells (Vero or Vero E6) were lysed in RIPA buffer (50 mM Tris-HCl pH 7.2, 150 mM NaCl, 0.1% SDS, 1% sodium deoxycholate, and 1% Triton X-100), which was supplemented with protease inhibitors. The lysate was then sonicated on ice for 5–10 s, and cleared by centrifugation at 17,000 *g* for 10 min at 4 °C. The cleared lysate-proteins were separated by SDS-PAGE and blotted on PVDF membrane (Carl Roth, Karlsruhe, Germany). Blots were blocked in Odyssey blocking buffer TBS (LI-COR Bioscience, Nebraska, USA) which was used to dilute the primary and secondary antibodies (indicated in the figure legends), except for Western blot in Figure 4e (1); here blocking of the membrane and dilution of the antibodies (K87 and sec. antibody) were done using Roti Block (Carl Roth, Karlsruhe, Germany). In Figure 4e (2 and 3) blocking was done using 5% non-fat milk in TBS-T and the dilution (1:10,000) of anti-WNV envelope antibody (GeneTex) and the secondary antibody was done using TBS-T. As secondary antibodies, IRDye^®^ 800CW anti-mouse or anti-rabbit (LI-COR Bioscience, Nebraska, USA, diluted 1:10000) were used. For detection, the Odyssey detection system (LI-COR Bioscience, Nebraska, USA) was used. 

### 2.5. Indirect Immunofluorescence Microscopy Analysis 

A549 cells (1.5 × 10^5^ cells/well in 12-well plate) were infected with ZIKV (the Asian or the African strain) using MOI = 0.1, or left uninfected. Vero E6 cells (0.375 × 10^5^ cells/well in 12-well plate) were infected with West Nile virus (WNV) using MOI = 1, or left uninfected. Forty-eight hours post infection, the cells were washed once with PBS and fixed for 15–30 min at RT with formaldehyde (Carl Roth, Karlsruhe, Germany) (4% in PBS). After washing with PBS, the cells were permeabilized for 10 min at RT with 0.5% Triton X-100 in PBS. The cells were washed again with PBS and blocked for 1 h at RT with 1% BSA in PBS solution, followed by incubation for 1 h at RT with the purified antibodies (1 µg/mL diluted in PBS) or 4G2 antibody (anti-flavivirus group antigen antibody from Merck, Darmstadt, Germany; diluted 1:300 in PBS). The cells were then washed with PBS for 30 min, followed by incubation for 1 h at RT with Cy3 anti-rabbit (Jackson Immunoreserearch, West Grove, USA; diluted 1:400 in PBS) or Alexa Fluor 488 anti-mouse (Thermo Fisher Scientific, Waltham, USA; diluted 1:1000 in PBS) secondary antibodies. For cell nuclei visualization, 0.1 µg/mL DAPI (Carl Roth, Karlsruhe, Germany) was added to the secondary antibody solution. Finally, the cells were washed with PBS for 30 min at RT and mounted on coverslips using 20 µL Mowiol (Merck, Darmstadt, Germany). Confocal laser scanning microscopy (CLSM 510; Carl Zeiss, Oberkochen, Germany) was used for visualizing the cell nuclei and the ZIKV/WNV E proteins.

### 2.6. Peptide Arrays for Determination of the Antibody Binding Sites

#### 2.6.1. Multipeptide Microarray Preparation

Peptide arrays (111 peptides), covering the first 454 residues of the ZIKV E protein (encompassing domains D1, D2, and D3), were synthesized and spotted onto microscope slides that were precoated with adhesive foil. The length of each peptide was 15 residues, and the overlapping length between the peptides was 11 residues (4 amino acid off-set). DMSO (Acros Organics via VWR, Darmstadt, Germany) was used for blank spots. Each peptide as well as DMSO spots were spotted in quadruplicates. The peptide synthesis and spotting of microarrays was done as previously described [23].

#### 2.6.2. Binding Sites Determination

The peptide microarrays were first incubated for 1 h at RT with blocking buffer (Odyssey blocking buffer TBS, LI-COR Bioscience, Nebraska, USA). The purified sera were diluted in blocking buffer to a final concentration of 0.3 µg/mL and were applied on the arrays for 1 h at RT, followed by washing for 30 min with TBS-T buffer. Anti-rabbit secondary antibody (IRDye^®^ 800CW anti-Rabbit, LI-COR Bioscience, Nebraska, USA) was diluted 1:10000 in the same blocking buffer and applied on the arrays for 1 h at RT, followed by washing for 30 min with TBS-T buffer. The arrays were then scanned by Odyssey detection system (LI-COR Bioscience, Nebraska, USA). For comparative quantification based on z-scores, the final signal for each peptide was calculated as following: (median signal of the quadruplicates of each peptide—median signal of the DMSO spots) / (standard deviation of the signal of DMSO spots).

### 2.7. Viruses

ZIKV French Polynesia PF13251013-18 and ZIKV Uganda 976 were kindly provided by Dr Musso (Institute Louis Malardé, Tahiti) and the European Virus Archive, respectively. Regarding infection with WNV, the isolate WNV NY99 was utilized. Propagation of ZIKV and WNV was performed in Vero and Vero E6 (African green monkey kidney cells) cells, respectively.

### 2.8. ZIKV Purification

Vero cells were infected (MOI = 0.2–1) with the French Polynesia PF13/251013-18 strain or the Uganda 976 strain of ZIKV (or left uninfected for control purification). Medium was changed 48 h after infection and five to seven days later, the supernatant was collected and cleared by centrifugation at 4600 *g* for 30 min at 4 °C, followed by ultracentrifugation at 150,000 *g* for 2.5 h at 4 °C. The pellet was then resuspended for 1 h at 4 °C in the following buffer: 0.12 M Tris-base, 0.06 M sodium acetate, 3 mM EDTA, pH 7.1, followed by centrifugation at 18,000 *g* for 10 min at 4 °C. One ml of the supernatant was applied on the top of discontinuous sucrose gradient prepared by dissolving sucrose in PBS as shown in Table 2:

The tubes were ultracentrifuged at 288,000 *g* for 16 h at 4 °C and eleven one-ml sucrose fractions were collected from the top of each tube and subjected to Western blot analysis.

### 2.9. ELISA

#### 2.9.1. Coating with Purified ZIKV E Proteins

The purified ZIKV E, ZIKV ED1+2, and ZIKV ED3 proteins were diluted (5 µg/mL) in 50 mM sodium carbonate coating buffer and applied overnight at 4 °C on ELISA plates (Corning Incorporated, NY, USA). The plates were then washed three times with PBS-T (PBS + 0.05% Tween20) and blocked with blocking solution (2% BSA in PBS) for 1 h at RT. Serial dilutions of the sera or the purified antibodies were applied for 2 h at RT, followed by washing three times with PBS-T. As secondary antibody, HRP-conjugated anti-rabbit IgG (GE Healthcare, Munich, Germany, diluted 1:1000 in 1% BSA in PBS-T,) was then applied for 1 h at RT, followed by washing three times with PBS-T. TMB ELISA substrate (Thermo Fisher Scientific, Waltham) was used for developing the plates and 1 N sulfuric acid (Carl Roth, Karlsruhe, Germany) was used to stop the reaction. For detection, the plates were read at 450 nm (TECAN infinite M1000, TECAN, Maennedorf, Switzerland).

#### 2.9.2. Coating with Purified Virus Particles

The purified viral particles (sucrose fractions number 10 diluted in coating buffer) were immobilized on ELISA plates. As a control, sucrose fraction number 10 from uninfected cells was applied as well. To ensure immobilizing equal amounts of viral particles from both strains, the amount of envelope protein, which was detected by Western blot in sucrose fraction number 10 from both strains, was used for normalization. After washing and blocking, serial dilutions of the purified antibodies were applied for 2.5 h at 37 °C. The secondary antibody was applied for 1 h at 37 °C, and ELISA procedure was continued as previously described. 

### 2.10. Plaque Reduction Neutralization Test (PRNT)

The sera were mixed in dilution series (1:20 to 1:320) with 200 pfu of ZIKV (Asian or African strain) or WNV for 1–2 h at 37 °C. 50–100 µL of the mixture was used to infect 3 × 10^5^ Vero cells (in case of ZIKV) and 3 × 10^5^ Vero E6 cells (in case of WNV) seeded in 6-well plates for 1–2 h at 37 °C. The medium was then removed and the cells were layered with 0.4% agarose solution (seaPlaque Agarose, Lonza, Basel, Switzerland). Three to six days later, the agarose layer was removed and the cells were fixed with formaldehyde solution (4% in PBS). The plaques were visualized by staining for 15 min at RT with crystal violet solution (0.1% in 20% ethanol, Merck, Darmstadt, Germany), followed by washing with distilled water. The plates were dried and the plaques were counted. As positive controls the neutralizing antibodies 4G2 and Z004-HRP (Absolute antibody) were used. In some experiments (indicated in the figure legends), the sera and the FBS, which was used to prepare the medium, were heat-inactivated for 30 min at 56 °C. In these experiments, DMEM high glucose medium (Biowest, Nuaillé, France) supplemented with 10% fetal bovine serum superior (Biochrom GmbH, Berlin, Germany), 2 mM l-glutamin (Merck, Darmstadt, Germany), 100 U/mL penicillin, and 100 µg/mL streptomycin was used.

### 2.11. Surface Plasmon Resonance (SPR)

The interaction between the purified sera and the different ZIKV envelope protein domains was analyzed by the SPR using the Biacore T200 system (GE Healthcare, Munich, Germany) at 25 °C. The purified ZIKV E, ZIKV ED1+2, and ZIKV ED3 proteins were immobilized using the amine coupling chemistry on a CM5 sensor chip (GE Healthcare, Munich, Germany) according to the manufacturer’s instructions to reach final responses of 121.3 RU, 123.5 RU, and 294.4 RU, respectively. Each of the purified sera was applied in five different concentrations: 12.5, 25, 50, 100, and 200 nM in a single cycle mode. The antibodies were diluted in the running buffer: PBS-T + 3 mM EDTA. The contact time, dissociation time, and flow rate were adjusted to 440–500 s, 2400 s, and 10 µL/min, respectively. The sensor surface was regenerated after each cycle by applying 3 × 30 s of 10 mM NaOH (30 µL/min). The unspecific binding to the blank-immobilized flow cell was subtracted. This unspecific binding might be due to the application of polyclonal antibodies as analyte. The experimental data were fitted in a 1:1 binding model using Biacore T200 evaluation software (GE Healthcare, Munich, Germany) to calculate the binding kinetics.

### 2.12. Software and Statistical Analyses

Analysis of the ELISA, peptide arrays, and the plaque reduction neutralization test (PRNT) data was done using GraphPad Prism 7 software. Illustration of the epitopes and binding regions was performed using PyMOL Molecular Graphic System, version 2.2.2. ELISA data, which are presented in Figure 2, Figure 3 and Figure 5, represent one of two independent experiments. PRNT data were collected from at least two independent experiments (if indicated one experiment) and are represented as mean ± SEM. SPR data were collected from two independent experiments.

## 3. Results

### 3.1. Production of Recombinant Proteins and Immunization of Rabbits

**Figure 1:**
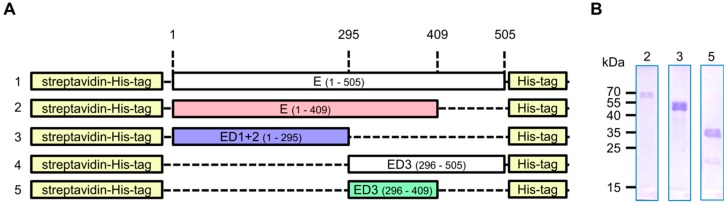
Construct design and protein production for rabbit immunization. (**A**) Schematic diagram of the designed constructs. The coding sequence of (1) the complete ZIKV E protein (aa 1–505), (2) the C-terminally truncated ZIKV E protein (aa 1–409), (3) the first two domains of ZIKV E protein (aa 1–295), (4) the Domain 3 of ZIKV E protein (aa 296–505), or (5) the C-terminally truncated Domain 3 of ZIKV E protein (aa 296–409) was cloned C-terminally to streptavidin-His-tag. Only constructs 2, 3, and 5 were successfully produced in *E. coli*; (**B**) Coomassie staining of SDS-gel loaded with purified and refolded proteins produced using the constructs 2, 3, and 5 (from **A**). The theoretical molecular weights of the purified proteins are: 60, 48, and 28 kDa, respectively.

In order to produce different domains of the ZIKV envelope protein (ZIKV E), five different bacterial expression plasmids were generated (Figure 1a). We aimed to produce (1) the complete ZIKV E protein (aa 1–505), which comprises the three domains (D1+2+3), the stem region, and the transmembrane domain; (2) the C-terminally truncated ZIKV E protein (aa 1–409), which lacks the stem region and the transmembrane domain; (3) the first two domains (D1+2) of ZIKV E protein (aa 1–295); (4) Domain 3 (D3) of ZIKV E protein (aa 296–505); and (5) the C-terminally truncated D3 of ZIKV E protein (aa 296–409). The plasmids were designed for the production of the various ZIKV E protein domains as fusion proteins with streptavidin. However, only constructs 2, 3, and 5 (which lack the stem region and the transmembrane domain) led to successful production of the recombinant proteins in *E. coli*. Purification of the recombinant proteins was successfully performed by isolation of inclusion bodies, followed by Ni-NTA affinity chromatography under denaturing conditions. The purity of the purified proteins was confirmed by Coomassie staining of SDS-gel (Figure 1b).

It was our aim to gain ZIKV-specific antibodies which recognize linear epitopes in the E protein, and to analyze their utility for the detection of denatured and native ZIKV E protein and correlate these properties with their potential neutralizing activity. For these reasons, denatured proteins were used for immunization. The purified and denatured proteins were used for rabbit immunization, in which two rabbits were immunized with each protein. Finally, six sera were collected and designated as shown in Table 3:

### 3.2. Generation and Purification of ZIKV E-Specific Antisera

**Figure 2:**
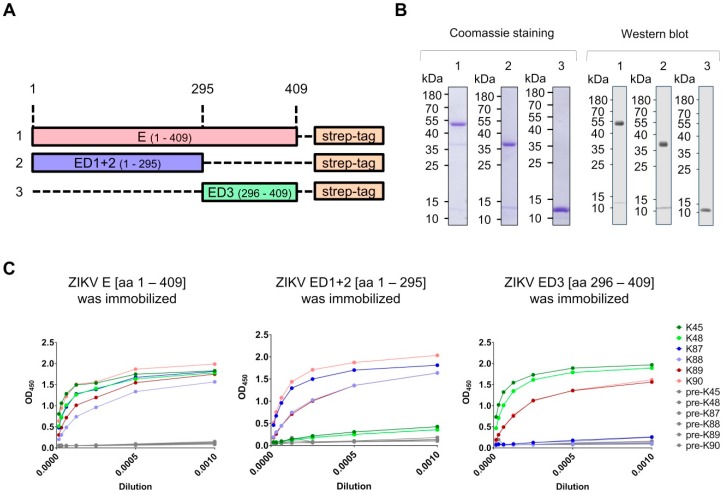
Determination of ZIKV E-specific antibodies in the sera. (**A**) Schematic diagram of the designed constructs, which were used for antibody characterization (and later for antibody purification). The constructs were designed for the production of strep-tagged-ZIKV E protein domains; (**B**) Coomassie staining (left) and Western blot (right) of SDS-gels loaded with the purified and refolded strep-tagged ZIKV envelope proteins: (1) ZIKV E, (2) ZIKV ED1+2, and (3) ZIKV ED3. The theoretical molecular weights of the purified proteins are: 46, 34, and 14 kDa, respectively. Anti-strep-tag (Novus, Wiesbaden, diluted 1:5000) and anti-mouse antibodies were used as primary and secondary antibodies, respectively; (**C**) Binding capacity of the rabbit sera to the different ZIKV E domains demonstrated by ELISA. ELISA plates were coated with the strep-tagged-ZIKV E domains and serial dilutions of the hyper-immune and the pre-immune sera were applied. The shown ELISA results represent one of two independent experiments.

To determine whether the collected sera contain ZIKV E domain-specific antibodies, the sera were tested for their reactivity against different ZIKV E domains using ELISA. The antigens, which were used for immunization, comprised ZIKV E domains as fusion proteins with streptavidin and His-tag. Therefore, using these antigens for the detection of ZIKV E-specific antibodies by ELISA was not possible due to the potential existence of streptavidin- and His-tag-specific antibodies in the collected sera. For this reason, three additional constructs, which allow the production of strep-tagged-ZIKV E protein domains without streptavidin and His-tag, were generated (Figure 2a). The production of these proteins was performed in *E. coli*, and the purification was achieved through inclusion body isolation and subsequent strep-tactin affinity chromatography. The identity and purity of the isolated proteins were proven by Western blotting and Coomassie staining of SDS-gel (Figure 2b). The purified and refolded proteins were immobilized on ELISA plates and the hyper-immune sera were applied in serial dilutions. As controls, the pre-immune sera were applied. The results showed that all hyper-immune sera contain ZIKV E-specific antibodies, and that these antibodies are directed to the corresponding domains of the E protein: Sera K89/K90 showed reactivity to ZIKV E protein, K87/K88 showed reactivity to ZIKV ED1+2 protein, and K45/K48 showed reactivity to ZIKV ED3 protein (Figure 2c). These data indicate that rabbit immunization with denatured ZIKV E proteins allows the successful production of ZIKV E domain-specific antibodies, which are capable of binding the refolded ZIKV E protein domains.

**Figure 3:**
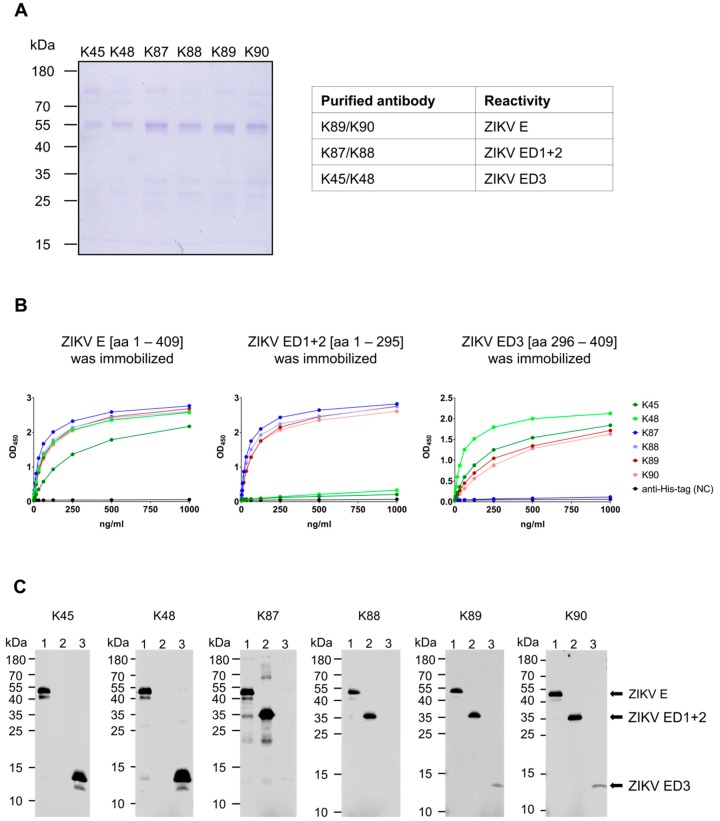
Purification of the ZIKV E domain-specific antibodies. (**A**) Coomassie staining of SDS-gel loaded with the purified antibodies; (**B**) Binding capacity of the purified antibodies to the different ZIKV E domains demonstrated by ELISA. ELISA plates were coated with the strep-tagged-ZIKV E domain proteins and serial dilutions of the purified antibodies were applied. Rabbit-derived His-tag-specific antibody was applied as negative control. The ELISA results represent one of two independent experiments; (**C**) Western blot analysis of SDS-gels loaded with the strep-tagged ZIKV envelope proteins: (1) ZIKV E, (2) ZIKV ED1+2, and (3) ZIKV ED3. The theoretical molecular weights of the purified proteins are: 46, 34, and 14 kDa, respectively. Each of the rabbit purified antibodies (K45–K90, 0.5 µg/mL) was used separately as a primary antibody. As secondary antibody, anti-rabbit was applied.

In order to isolate the ZIKV-specific antibodies from the sera, affinity beads were used. The purified strep-tagged-ZIKV E protein domains were coupled to NHS-activated agarose beads and were used to isolate envelope domain-specific antibodies from the corresponding rabbit sera as following: Beads coupled with ZIKV E protein were used for purification of sera K89 and K90. Beads coupled with ZIKV ED1+2 protein were used for purification of sera K87 and K88. Beads coupled with ZIKV ED3 protein were used for purification of sera K45 and K48. Purification was done using ÄKTA purifier system. The success of the purification procedure was proven by applying the eluted antibodies on SDS-gel and further Coomassie staining (Figure 3a). The specific reactivity of the isolated antibodies to the corresponding ZIKV E domains was tested and proven by ELISA (Figure 3b). In addition, the specific reactivity was also tested by subjecting the purified proteins (ZIKV E, ZIKV ED1+2, and ZIKV ED3) to Western blot analysis using the purified antibodies (K45–K90) as primary antibodies (Figure 3c). This shows that purification of ZIKV E domain-specific antibodies was done successfully using the corresponding ZIKV E domains.

### 3.3. Recognition of Denatured ZIKV E Protein from the Asian and the African Virus Strains by the Purified Antibodies

**Figure 4:**
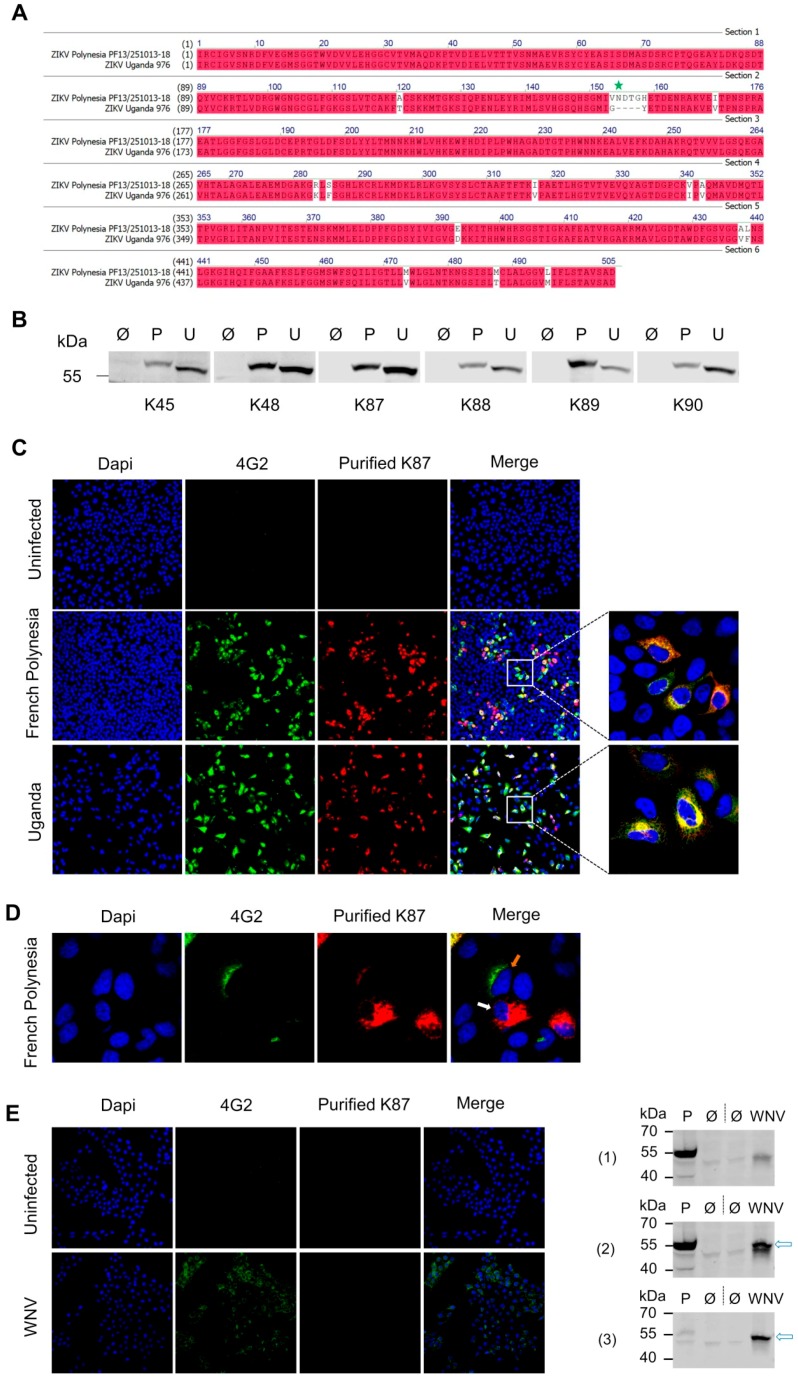
Recognition of ZIKV E protein in infected cells by the purified antibodies. (**A**) Alignment of the amino acid sequence of ZIKV E protein from the Asian (French Polynesia PF13251013-18) and the African strain (Uganda 976). Identical residues are highlighted in red, and the single glycosylation site in ZIKV E protein (N154) in the Asian strain-E protein is marked with green star. Alignment was done using Vector NTI; (**B**) Western blot analysis of lysates from A549 cells, which were either infected with ZIKV French Polynesia (P) or Uganda (U) strains, or left uninfected (Ø). The purified antibodies (K45–K90, 0.2–0.5 µg/mL) were applied as primary antibodies. Anti-rabbit was applied as secondary antibody; (**C**) Confocal laser scanning microscopy (CLSM) pictures of infected (or uninfected) and fixed A549 cells stained with purified K87 antibody. Staining with anti-flavivirus group antigen antibody (4G2) was performed for comparison; (**D**) CLSM pictures of infected and fixed A549 cells which were stained like in C. The white arrow indicates a cell with a strong staining with K87 antibody and almost no staining with 4G2 antibody. The orange arrow indicates a cell with a strong staining with 4G2 antibody and very weak staining with K87 antibody; magnification 16× or 100× was applied; (**E**) CLSM pictures (left) and Western blot analysis (right) of WNV-infected (or uninfected) Vero E6 cells stained with purified K87 antibody. For the immunofluorescence microscopy, staining with anti-flavivirus group antigen antibody (4G2) served as positive control. For Western blot, ZIKV (Asian strain)-infected or uninfected Vero cell lysates were applied as positive and negative controls, respectively. The membrane was incubated with purified K87 antibody (1) (0.2 µg/mL) followed by incubation with anti-WNV envelope antibody (2) (GeneTex, diluted 1:10,000, incubation for 2 h at RT), or the membrane was directly incubated with WNV envelope antibody (3). For both K87 and anti-WNV envelope antibodies, anti-rabbit was applied as secondary antibody. The theoretical molecular weight of WNV envelope protein is 54 kDa (indicated with arrows).

The amino acid sequence of the ZIKV E protein is highly conserved between the Asian (ZIKV French Polynesia = P) and the African (ZIKV Uganda = U) strains (Figure 4a), however, not identical. Since the sequence of the Asian strain was considered to generate the antigens, which were used for immunization, we were interested in investigating whether the purified antibodies can recognize ZIKV E protein from both the Asian and the African strains. For this, A549 cells were infected with either of these two strains, and Western blotting and immune fluorescence staining techniques were applied. The results showed that specific staining for both virus strains was obtained using each of our purified antibodies (Figure 4b,c), which demonstrates the capacity of these antibodies to recognize denatured ZIKV E proteins from both strains. In Figure 4, the fluorescence microscopy pictures of A549 cells, which were infected with the Asian (French Polynesia) or the African (Uganda) strains and were stained with the purified K87 antibody (specific for ZIKV ED1+2), are shown as an example. Comparable results were obtained using the other purified antibodies (K48, K88, K89, and K90), except for the purified K45 antibody, where less intense staining was observed. For comparison, the cells were co-stained with a flavivirus group-specific antigen specific antibody (4G2). When purified K87 and 4G2 antibodies were used together for staining, almost all infected cells showed double staining with these two antibodies, and only a few cells were stained with only one antibody (Figure 4d). This might be explained by the difference in the detection levels between our purified antibodies and 4G2 antibody, and/or by different recognition of ZIKV E protein degradation products from these different antibodies.

Since reactivity of ZIKV-specific antibodies with other related *Flaviviruses* is considered to be problematic with respect to the development of ZIKV-specific diagnostic tools, the potential cross-reactivity of the antibodies (K45–K90) with WNV, as a related flavivirus, was analyzed. For this reason, Vero E6 cells were infected with WNV, or left uninfected, and the purified antibodies (K45–K90) were applied to check their capacity to react with WNV E proteins using Western blot and immunofluorescence microscopy (Figure 4e). Staining with purified K87 was shown here as an example, and similar staining results were obtained using the other purified antibodies. The results indicate that these antibodies were not able to detect WNV E protein from infected Vero E6 cells, arguing against a cross-reactivity with the envelope protein of a related flavivirus.

These data and the ELISA data (Figure 3) demonstrate the capacity of our purified antibodies to detect both the denatured and the native ZIKV E protein, and that these antibodies are suitable for in-detail analysis of ZIKV infection using Western blot and immunofluorescence microscopy techniques. In addition, our antibodies showed no reactivity against denatured WNV envelope protein, as one of the related *Flaviviruses*.

### 3.4. Binding of the Purified Antibodies to the Surface of ZIKV Particles

**Figure 5:**
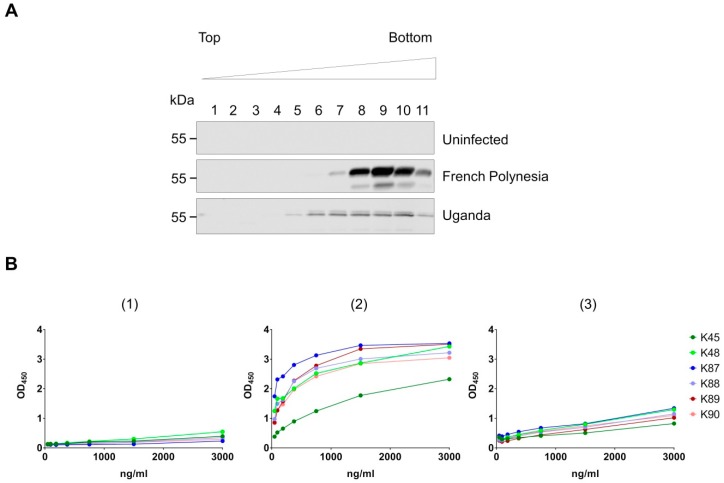
Binding of the purified antibodies to the surface of ZIKV. (**A**) Culture supernatant from uninfected (Ø) or infected Vero cells with the Asian (French Polynesia) or the African (Uganda) strain was collected and ultracentrifuged. The pellet was resuspended and loaded on discontinuous sucrose gradient followed by ultracentrifugation and analysis of the collected sucrose fractions by Western blot. The purified antibody K87 (specific for ED1+2) was used as primary antibody, and anti-rabbit was applied as secondary antibody; (**B**) The sucrose fraction number 10 from uninfected cells (1), from the Asian strain-infected cells (2), or from the African strain-infected cells (3) were used for immobilization on ELISA plates. The ELISA plates were incubated with serial dilutions of the purified antibodies (K45–K90), followed by incubation with secondary antibodies, substrate, and detection.

Next, we wanted to investigate whether the purified antibodies bind the surface of intact ZIKV particles. To achieve this, ZIKV particles were first enriched from supernatant collected from infected Vero cells using ultracentrifugation and sucrose density gradient centrifugation (Figure 5a). Supernatant from uninfected cells was treated similarly as control. Next, the enriched virus particles were immobilized on ELISA plates to test the binding capacity of the purified antibodies to intact viral particles. The results showed that all antibodies bind the immobilized virus to different extent (Figure 5b). Interestingly, the binding to the African strain-derived ZIKV was weaker as compared to the Asian strain-derived particles, although comparable amounts of enriched particles were immobilized in both cases. These results show the capacity of our purified antibodies to recognize and bind surface-exposed epitopes on the intact ZIKV particles.

### 3.5. Binding Sites Determination of the Purified Antibodies

**Figure 6:**
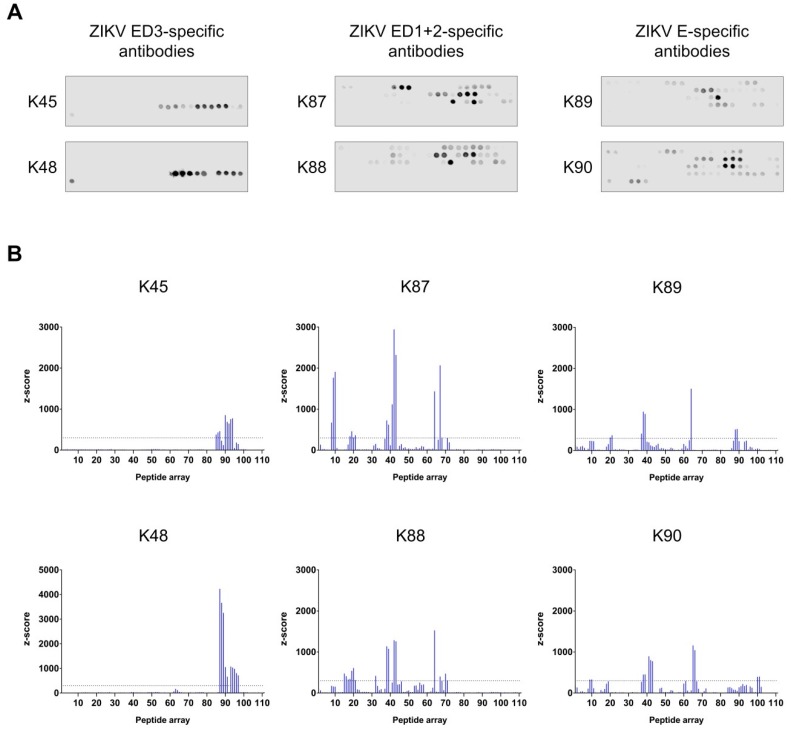
Binding sites determination of the purified antibodies. (**A**) The arrays with spotted peptides were incubated with the purified antibodies (K45–K90), followed by incubation with secondary antibody and scanning; (**B**) The results from the peptide arrays (in **A**) are illustrated on graphs in which the peptide number is shown on the X-axis and the z-score is shown on the Y-axis. The dotted lines represent the cut-off.

In order to determine the binding sites of our purified antibodies, multipeptide microarrays covering the first 454 residues of the E protein (the ectodomain plus the stem region) were generated. In total, 111 peptides (15-mer, 4 aa off-set) were synthesized and named P1 to P111. The arrays with spotted peptides were incubated with each of the purified antibodies (K45–K90), followed by incubation with secondary antibodies and detection (Figure 6a). The finally calculated signal intensities (z-scores) of the peptides are shown in Figure 6b. Statistically, z-score above 3 is considered as a relevant signal over the background; however, to focus on the most dominant antibody binding capacities, z-score of 300 was taken as a cut-off to define positive binding. In some cases, the peptides were recognized in a similar (not identical) pattern from the different antibodies. In such cases, the similar areas were clustered as sequential stretches in order to define the binding sites. For example, P8–P10 and P9–P10 were positive (above the cut-off) in the arrays incubated with K87 and K90 antibodies, respectively; therefore, the binding site was in this case considered to contain the peptides P8–P10. In total, seven main binding sites were determined as following: Immunization with ZIKV ED3 resulted in antibodies (K45 and K48) which bind to the peptides 85 to 97 (P85–P97) on the peptide arrays. These peptides correspond to residues 337–399 in the ZIKV E protein and are located in the D3 domain. Immunization with ZIKV ED1+2 resulted in antibodies (K87 and K88) which bind to sites distributed in the D1+2 domain and cover the following residues: 29–51 (P8–P10), 57–95 (P15–P21), 125–139 (P32), 145–183 (P37–P43), and 253–295 (P64–P71). Immunization with ZIKV E resulted in antibodies (K89 and K90) which showed binding sites distributed all over the three domains covering the following residues: 29–51 (P8–P10), 57–95 (P15–P21), 145–183 (P37–P43), 253–295 (P64–P71), 337–399 (P85–P97), and 397–415 (P100–P101).

### 3.6. Surface Exposure of the Binding Sites

**Figure 7:**
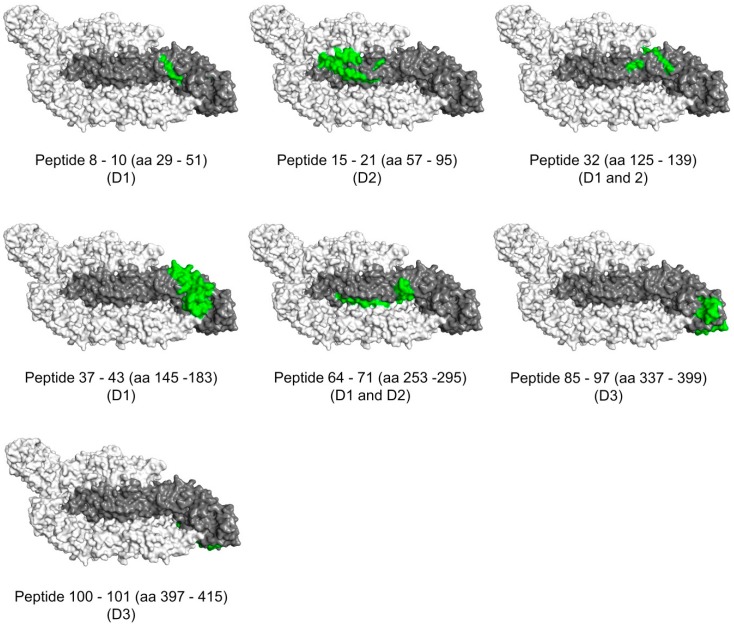
Surface exposure of the binding sites. The residues which are included in each of the defined binding sites of the purified antibodies (K45–K90) are highlighted in green on the surface representation (looking from outside the virion) of the ZIKV E protein structure [modified from PDB ID: 5IRE]. The binding sites with the corresponding amino acid number and the E domain are indicated below each representation. In each representation, single ZIKV E monomer is shown in gray and the surrounding two E monomers are shown in white.

The data from the peptide arrays allowed us to identify linear binding sites/epitopes of the purified antibodies without, however, providing further information about their topology. Therefore, we were interested in investigating whether these sites are surface-exposed on the mature ZIKV particles. To achieve this, we mapped the determined binding sites (from Figure 6) on the resolved ZIKV protein structure (PDB ID: 5IRE) (Figure 7). In Figure 7, the surface representation (looking from outside the virion) of one ZIKV E protein monomer (colored in gray) and two neighboring E monomers (colored in white) is shown. The residues covered by each binding site (determined using the peptide arrays, Figure 6), were marked in green on the central E monomer. This representation allowed us to define whether each binding site of our purified antibodies is surface exposed and thus facing outside the virion, embedded between two monomers, or facing the inner side of the virion.

As shown in Figure 7, two binding sites are mostly exposed on the surface of the virus particles: The first site covers the residues 57–95 (P15–P21) and is recognized from the ED1+2-specific antibodies (K87 and K88) and from the E-specific antibody K89. The second site covers the residues 145–183 (P37–P43) and is recognized from all antibodies except the ED3-specific antibodies (K45 and K48). In addition, four binding sites seem to be partially exposed: Residues 29–51 (P8–P10) which are recognized from the ED1+2-specific antibody K87 and E-specific antibody K90, residues 125–139 (P32) which are recognized from the ED1+2-specific antibody K88, residues 253–295 (P64–P71) which are recognized from all antibodies except the ED3-specific antibodies, and residues 337–399 (P85–P97) which are recognized from both ED3-specific antibodies (K45 and K48) and E-specific antibody K89. The remaining binding site (P100–P101), which covers the residues 397–415 and is recognized from the E-specific antibody K90, seems not to be surface exposed.

Table 4 summarizes the recognized sites with the corresponding E domains, the amino acid sequences, and the surface exposure.

### 3.7. Binding Affinity to the ZIKV E Domains

**Figure 8:**
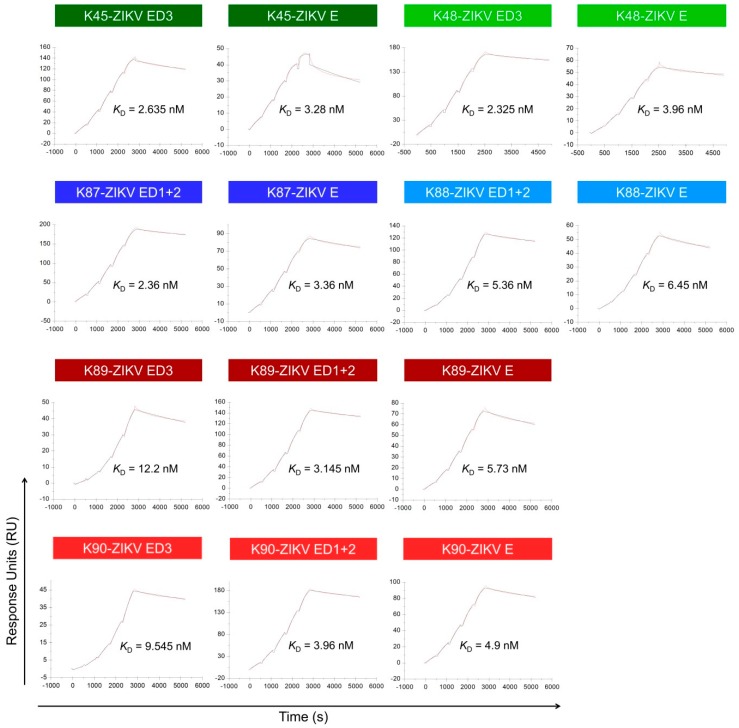
SPR analysis of the interaction between the purified antibodies and ZIKV E domains. The purified ZIKV E domains were immobilized on CM5 chip and the purified sera were applied in dilution series (12.5–200 nM) using a single cycle mode. The background binding to the blank-immobilized flow cell was subtracted, and the experimental data were fitted in a 1:1 binding model. The experimental data are shown in red lines and the fitted data in black lines. Sensograms from one of two independent experiments are shown. The shown *K*_D_ values are mean values of the two independent experiments.

For further characterization, we analyzed the binding kinetics of the purified antibodies (K45–K90) to the different ZIKV E domains using surface plasmon resonance (SPR) on a Biacore system (Figure 8 and Table 5). For this purpose, we immobilized purified ZIKV E, ZIKV ED1+2, and ZIKV ED3 proteins on CM5 chip and analyzed the interaction between each of the purified antibodies and the immobilized proteins. Regarding ED3-specific antibodies (K45 and K48), the recorded *K*_D_ values for K45-ED3 and K48-ED3 were 2.635 nM and 2.325 nM, respectively. These two antibodies showed slightly decreased affinity to the complete E protein with *K*_D_ values of 3.28 nM and 3.96 nM, respectively. The *K*_D_ values for the ED1+2-specific antibodies (K87 and K88) were 2.36 nM for K87-ED1+2, 3.36 nM for K87-E, 5.36 nM for K88-ED1+2 and 6.45 nM for K88-E. Finally, the E-specific antibodies (K89 and K90) showed, as expected, binding to each of the complete E, ED1+2, and ED3. The measured *K*_D_ values for these two antibodies were: 5.73 nM for K89-E, 3.145 nM for K89-ED1+2, 12.2 nM for K89-ED3, 4.9 nM for K90-E, 3.96 nM for K90-ED1+2, and 9.545 nM for K90-ED3. In summary, all purified antibodies showed high affinity to the corresponding ZIKV E domains. Consistent with the ELISA data, no binding of the ED3-specific antibodies (K45 and K48) to the ED1+2 was observed. Similarly, no binding of the ED1+2-specific antibodies (K87 and K88) to the ED3 was observed.

### 3.8. Neutralizing Activity of the Purified Antibodies

**Figure 9:**
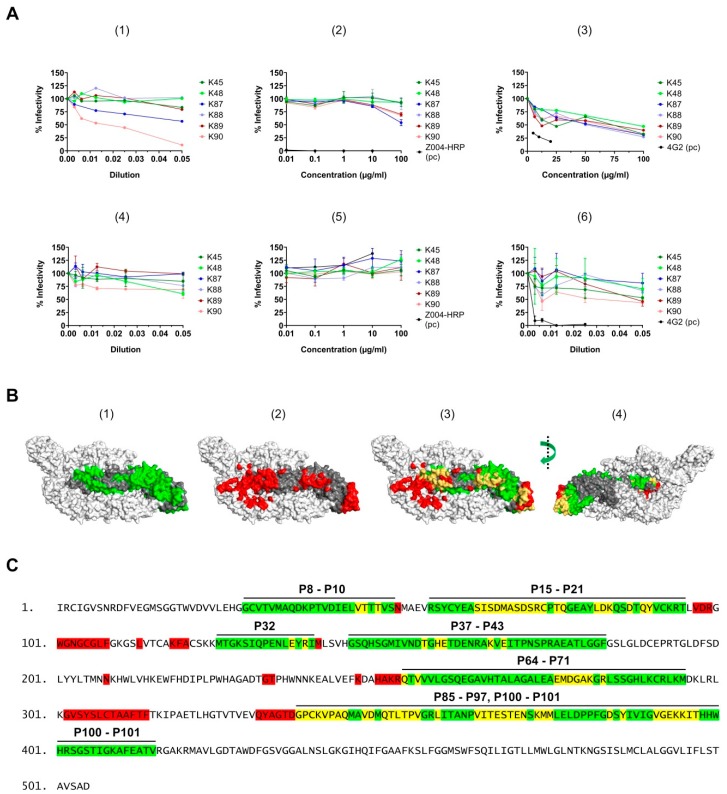
Correlation between the neutralizing activity and the binding sites. (**A**) Serial dilution of the sera (in 1, 4, and 6) or the purified antibodies (in 2, 3, and 5) was incubated with the Asian strain (in 1, 2, and 6) or the African strain (in 3) of ZIKV, or with WNV (in 4 and 5). The serum/purified antibody-virus mixture was used to infect Vero cells (in case of ZIKV) or Vero E6 cells (in case of WNV). The neutralizing antibody 4G2 or Z004-HRP (anti-ZIKV E Domain 3, Absolute antibody) were used as controls, however, failed to neutralize WNV. For quantification, the results are presented as % infectivity, and the number of plaques in the control sample, where the virus was applied without previous mixing with serum, was set as 100% infectivity. Data are represented as mean ± SEM. The dilution fold of the serum (in 1, 4, and 6) and the antibody concentration (µg/mL, in 2, 3, and 5) are indicated in each case. The serum was applied either with (in 1) or without (in 4 and 6) heat-inactivation. The results were collected from two independent experiments, except for Uganda (in 3) and Polynesia (in 1) from one experiment; (**B**) (1) All binding sites, which were marked in Figure 7, are shown here in green color on a single ZIKV E molecule. (2) Footprints of 14 ZIKV neutralizing antibodies are shown in red on the surface representation of ZIKV E protein. The footprints are for the following antibodies: ZK2B10, ZK-48, ZK-64, ZK-67, Z006, Z20, Z3L1, Z021, C8, C10, A11, 2A10G6, ZKA190, and ZIKV-195. (3) and (4) The defined binding sites of our antibodies (green) and the footprints of the neutralizing antibodies (red) are marked on the same ZIKV E molecule. The overlapping residues are colored in yellow. The central E molecule is colored gray and the adjacent two E molecules are colored white. [Modified from PDB ID: 5IRE]; (**C**) The same color representation as in B is applied on the amino acid sequence of the ZIKV E protein (PF 13/251013-18). The peptide numbers (from the arrays), which correspond to the defined binding sites, are shown above the sequence.

To correlate the obtained data for the purified antibodies (K45–K90) with their potential neutralizing activity, we performed plaque reduction neutralization test (PRNT) using the Asian and the African strains of ZIKV. Both the sera and the purified antibodies were applied in these experiments. As control, this experiment was performed with heat-inactivated sera (in case of the Asian strain). Interestingly, the data showed that the sera and the purified antibodies were not able to potently neutralize ZIKV infection under the applied conditions (Figure 9a). In some cases, a weak neutralization was observed; however, this was only the case when high concentrations (1:20 dilution) of the antibodies or the sera were applied. Therefore these antibodies were considered as week/non-neutralizing for ZIKV. In addition, the neutralizing capacity of the sera and of the purified antibodies against WNV was studied. The data indicate that the antibodies have also week/non- neutralizing activity against WNV (Figure 9a). These results indicate that our antibodies are weak/none neutralizers and that the defined binding sites might represent non-/weak-neutralizing epitopes. In order to compare the binding sites of our antibodies (K45–K90) with the defined epitopes of ZIKV E neutralizing antibodies, we marked (shown in red) the footprints of 14 ZIKV E neutralizing antibodies on the ZIKV E structural model and compared them to the binding sites that we defined for our antibodies (shown in green) (Figure 9b). Clear definition of the epitope, as well as exhibiting neutralizing activity, were considered as prerequisites for the selection of these 14 neutralizing antibodies: ZK2B10 [12], ZK-48 [13], ZK-64 [13], ZK-67 [13], Z006 [16], Z20 [15], Z3L1 [15], Z021 [17], C8 [11], C10 [20], A11 [11], 2A10G6 [10], ZKA190 (epitope identified by NMR, [14]), and ZIKV-195 [18]. The footprints of Z23 [15] and ZIKV-117 [19] were not included here due to relatively low resolution (Z23) and difficulties in depicting the epitope residues for either the two-fold or three-fold axes (ZIKV-117). Compared to the other mentioned antibodies, ZK2B10 is considered to have relatively lower neutralizing capacity, but this antibody was included in the analysis, since it is the only one which specifically binds to the fusion loop of ZIKV E protein. Detailed comparison revealed only a partial overlap (shown in yellow) between our defined binding sites and ZIKV neutralizing epitopes (Figure 9c,d). Higher degree of overlapping was observed in the sites P15–P21 (aa 57–95) and P85–P97 (aa 337–399), while other sites seemed to have lower overlapping with the neutralizing epitopes. These data indicate that the binding sites of the purified antibodies (K45 to K90) might contain non-/weak-neutralizing epitopes.

## 4. Discussion

In this study, we present the generation and characterization of ZIKV E-specific polyclonal antibodies, for which we compared the binding sites and the neutralizing activity with the previously described ZIKV neutralizing antibodies. For antibody production, we immunized rabbits with different domains of ZIKV E protein, which were produced in *E. coli*. The production of ZIKV E domains in *E. coli* and the further immunization was already described in other studies in the literature (examples are References [24], [10], [25], [13]). Aiming to produce domain-specific antibodies, we used each of the truncated ZIKV E protein (aa 1–409), ZIKV E domains 1+2 (ZIKV ED1+2: aa 1–295) and the truncated ZIKV E Domain 3 (ZIKV ED3: aa 296–409) in their denatured form for immunization. Truncation (deletion of the C-terminal stem region and the transmembrane domain) was performed to ensure efficient production of the recombinant proteins in *E. coli*. Since the antigens were used in their denatured form for immunization, the produced antibodies were expected to recognize sequential epitopes. Indeed, all our produced and purified antibodies were able to recognize the denatured ZIKV E protein from infected cells using Western blot (Figure 4). Interestingly, the molecular weight of the detected E protein from cells infected with the Asian strain was slightly higher compared to the African strain (Figure 4). This might be partially explained by the single glycosylation, which occurs on N154 in the Asian strain E protein, and is absent in the African strain [26]. Additionally, four residues in E protein of the Asian strain, which are missing in the African one, might contribute to the observed shift. Importantly, to purify the antibodies from the sera, we used purified ZIKV E protein domains in their native form; therefore, all purified antibodies were expected to recognize sequential epitopes, which are surface-accessible on the soluble, folded ZIKV E protein domains. ELISA data (Figure 3b), Western blot data (Figure 3c), and SPR data (Figure 8) showed indeed that the purified antibodies bind, in a domain-specific pattern, the soluble ZIKV E protein domains. Moreover, ELISA data in Figure 5 show the capacity of our antibodies to bind the surface of intact immobilized ZIKV particles. Interestingly, the measured binding to the Asian strain-particles was stronger than the binding to the African strain-derived particles. This difference might be partially explained by the difference in the amino acid sequence between the two strains. The sequence between aa 145 and 183 in the Asian strain-E protein contains four residues, N154, D155, T156, and G157, which are missing in the Uganda strain-E protein. This sequence belongs to Domain 1 and is recognized from the purified antibodies isolated from the sera K87, K88, K89, and K90. In addition, aa 337 to 399, which are recognized from the ED3-specific antibodies (K45 and K48) and the E-specific antibody K89, contain three substitutions between the two strains: A343V, V341I, and E393D. These differences might explain, to a limited extent, the higher binding capacity to the French Polynesia strain particles. However, since the remaining defined binding sites are mainly conserved, the observed difference is unlikely to be mainly strain-related. Other possibilities, such as lower immobilizing efficiency and lower stability of the African strain purified particles, might be causative for the observed results. Binding of our antibodies to the surface of ZIKV particles (at least to the Asian strain) indicates that the recognized epitopes are not only surface-exposed in the soluble form of ZIKV E proteins, but also in the context of the intact virus. Additional methods, such as co-immunoprecipitation, can also be further applied to support these findings. Peptide array results allowed us to identify seven binding sites of our antibodies in the ZIKV E protein (Figure 6). These sites are distributed among the three E domains, and are, in some cases, recognized to different extents from the different antibodies. Mapping of these sites on the resolved structure of ZIKV revealed that some of these sites seem to be partially or not surface-exposed on the virus surface (Figure 7). The site P64–P71 is located partially in the interface between two E monomers, and the site P100–P101 faces the inner side of the virion (Figure 7). These sites do not seem to be fully surface-exposed; however, the conformational dynamics of E protein (flavivirus E protein in general) might play a role in exposing such cryptic epitopes on the surface of ZIKV particles [27]. Interestingly, the PRNT data suggest that our antibodies are not able to potently neutralize ZIKV infection in vitro under the applied conditions (Figure 9). These results cannot be explained by low affinity binding, since SPR data showed high binding affinity with *K*_D_ in the range of nM to the corresponding ZIKV E protein domains. We suppose that the determined binding sites of our antibodies might contain non-/weak-neutralizing epitopes in the ZIKV E protein. Indeed, comparison between the binding sites, which were defined in this study, and the clearly defined epitopes of ZIKV neutralizing antibodies revealed only partial overlap, which may support our assumption. This indicates that the neutralizing epitopes are exactly defined. Therefore, our data contribute to increase our knowledge about the distribution pattern of neutralizing and non-/weak-neutralizing epitopes. Even the knowledge about non-/weak-neutralizing epitopes will be helpful for the development of robust test systems for the detection of protective antibodies. In light of the frequent fatal outcome of ZIKV infection during pregnancy, the development of test systems to determine the existence/absence of protective immune status is of major relevance. Cross-reactivity antibodies raised against ZIKV with other *Flaviviruses*, such as Dengue virus and WNV, is one of the difficulties in the development of ZIKV-specific diagnostic tools. The presented data suggested that the antibodies generated and characterized in this study do not react with WNV E protein in Western blot analysis and immunofluorescence microscopy (Figure 4) and exhibit weak/no neutralizing activity against WNV (Figure 9). These results argue against a general cross-reactivity of these antibodies with other *Flaviviruses* as we have excluded the reactivity of our antibodies against one of ZIKV-related *Flaviviruses*, as WNV, therefore, the presented information might be helpful to improve specificity of ZIKV-detection tools. Testing the reactivity of the antibodies (K45–K90) against other related *Flaviviruses*, such as Dengue virus, should be considered in further work to extend the information about ZIKV-specificity of these antibodies.

The fatal outcome of ZIKV infection during pregnancy requires a detailed knowledge about neutralizing and non-neutralizing epitopes for the development of robust detection systems that are able to discriminate between just cross-reactive and protective antibodies. The identification of epitopes that are not the base of a neutralizing immune response helps to draw a map showing the distribution of neutralizing and non-neutralizing epitopes. This could contribute to the development of assay systems that selectively determine protective antibodies.

In summary, we describe in this work the production of ZIKV E domain-specific antibodies, which bind, with high affinity, to sites distributed among the three E domains. These sites overlap only partially with the previously defined neutralizing epitopes of ZIKV which might explain why the purified antibodies have weak/no neutralizing activity. The information presented in this study helps to extend knowledge of the antigenicity of ZIKV E protein and the relationship between epitopes and the neutralizing activity to improve the detection assays and the selection of new candidates for vaccine development.

## Figures and Tables

**Table 1:** ZIKV E domain selection for sera purification

ZIKV E Protein Domain (S)	Serum
ZIKV E protein (aa 1–409)	K89 and K90
ZIKV ED1+2 protein (aa 1–295)	K87 and K88
ZIKV ED3 protein (aa 296–409)	K45 and K48

**Table 2:** Composition of the sucrose gradient which was used for ZIKV purification.

10% Sucrose	20% Sucrose	30% Sucrose	40% Sucrose	50% Sucrose	60% Sucrose
2 mL	1.5 mL	1.5 mL	1.5 mL	1.5 mL	1.5 mL

**Table 3:** Designation of the sera after rabbit immunization with ZIKV E protein domains.

Protein Used for Immunization	Serum
Streptavidin-ZIKV E (aa 1–409)	K89/K90
Streptavidin-ZIKV E domains 1+2 (aa 1–295)	K87/K88
Streptavidin-ZIKV E Domain 3 (aa 296–409)	K45/K48

**Table 4:** Detailed information about the determined binding sites of the purified antibodies.

Peptide	Amino Acid	Corresponding Sequence	Domain	Recognition	Surface Exposure
**P8–P10**	29–51	GCVTVMAQDKPTVDIELVTTTVS	1	K87, K90	partially
**P15–P21**	57–95	RSYCYEASISDMASDSRCPTQGEAYLDKQSDTQYVCKRT	2	K87, K88, K89	mostly
**P32**	125–139	MTGKSIQPENLEYRI	1 and 2	K88	partially
**P37–P43**	145–183	GSQHSGMIVNDTGHETDENRAKVEITPNSPRAEATLGGF	1	K87, K88, K89, K90	mostly
**P64–P71**	253–295	QTVVVLGSQEGAVHTALAGALEAEMDGAKGRLSSGHLKCRLKM	1 and 2	K87, K88, K89, K90	partially
**P85–P97**	337–399	GPCKVPAQMAVDMQTLTPVGRLITANPVITESTENSKMMLELDPPFGDSYIVIGVGEKKITHH	3	K45, K48, K89	partially
**P100–P101**	397–415	THHWHRSGSTIGKAFEATV	3	K90	no

**Table 5:** *K*_D_ values for the binding of the purified antibodies to the different ZIKV E domains.

	Binding to ZIKV ED3		Binding to ZIKV ED1+2		Binding to ZIKV E	
Antibody	*K*_D_ (nM)	Mean Value of *K*_D_ (nM)	*K*_D_ (nM)	Mean Value of *K*_D_ (nM)	*K*_D_ (nM)	Mean Value of *K*_D_ (nM)
**K45**	2.93	2.635	-	-	4.04	3.28
	2.34		-		2.52	
**K48**	1.79	2.325	-	-	2.96	3.96
	2.86		-		4.96	
**K87**	-	-	2.43	2.36	3.25	3.36
	-		2.29		3.47	
**K88**	-	-	4.85	5.36	6.06	6.45
	-		5.87		6.84	
**K89**	11.3	12.2	3.2	3.145	5.8	5.73
	13.1		3.09		5.66	
**K90**	9.42	9.545	3.13	3.96	3.85	4.9
	9.67		4.79		5.95	
